# Prevalence of Tinnitus and Its Associated Factors in the PERSIAN Guilan Cohort Study

**DOI:** 10.22038/IJORL.2022.63809.3187

**Published:** 2023-01

**Authors:** Rasool Panahi, Mir Mohammad Jalali, Farahnaz Joukar, Saman Maroufizadeh, Mohammadreza Naghipour, Fariborz Mansour-Ghanaei

**Affiliations:** 1 *Otorhinolaryngology Research Center, School of Medicine, Guilan University of Medical Sciences, Rasht, Iran. *; 2 *Gastrointestinal and Liver Diseases Research Center,* * Guilan University of Medical Sciences, Rasht, Iran. *; 3 *Department of Biostatistics, School of Nursing and Midwifery, Guilan University of Medical Sciences, Rasht, Iran. *

**Keywords:** Cohort study, Prevalence, Tinnitus

## Abstract

**Introduction:**

Tinnitus can be associated with many auditory and non-auditory factors, and its prevalence varies widely in the literature. There is no large sample of published data on tinnitus prevalence and its associated factors in Iran. Here, we analyzed the PERSIAN Guilan Cohort Study (PGCS) data and reported the prevalence of tinnitus and some of the risk factors related to tinnitus in the Iranian population.

**Materials and Methods:**

This cross-sectional study was conducted on 10520 men and women between 35 and 70 years old. The prevalence of tinnitus and associations between tinnitus and age, sex, habitat, marital status, employment status, socioeconomic status, educational level, lifestyle habits, and comorbid diseases were examined using simple and multiple logistic regression analyses.

**Results:**

The prevalence of tinnitus was 6.4% in this study. Based on the adjusted analysis, only older age (odds ratio: 2.60, 95% confidence interval: 1.88 – 3.60), residency in a rural area (odds ratio: 1.22, 95% confidence interval: 1.03 – 1.44), cigarette smoking (odds ratio:1.33, 95% confidence interval: 1.04 – 1.72), and having other comorbidities (odds ratio: 2.75, 95% confidence interval: 2.19 – 3.44) were related to tinnitus. In addition, the results of subgroup analyses by sex were mostly consistent with the overall analysis.

**Conclusions:**

Our results revealed that the prevalence of tinnitus in the north of Iran is comparable with other communities. Age and other comorbidities were among the most related factors to tinnitus.

## Introduction

Tinnitus is a perception of sound in one or two ears or the head without an external source and is a rather common complaint (1). The prevalence of tinnitus varies widely from 4.6 (2) to 46% (3) in different studies according to the definition of tinnitus, the study design, and the study population. About 30% of the general population have had at least one episode of tinnitus during their life, and about 6% develop severe tinnitus conditions (4). Tinnitus can be associated with many auditory and non-auditory factors, including hearing loss and noise exposure, age, sex and race, anxiety and depression, Body Mass Index (BMI), blood pressure, cardiac disease, smoking and alcohol (3). The association between tinnitus and hearing loss is well documented (5-7), but there is no consensus about many other factors related to the presence of tinnitus. For instance, in a large group of the noise-exposed population, Chung and colleagues reported no direct relationship between tinnitus with sex, age, and smoking. However, there was an indirect relationship (8). A study on Japanese elders showed no significant sex difference among tinnitus patients (9). Park and colleagues reported no association between smoking, alcohol drinking, and a history of anxiety with tinnitus (10). These divergent results suggest that there need to be more studies on factors related to tinnitus in different populations. Many non-auditory factors are associated with tinnitus, and knowledge about them can help better understand tinnitus. Cohort studies in large populations provide valuable information on the prevalence of medical conditions, including tinnitus. In the only available study on 3207 Iranian urban individuals, the prevalence of tinnitus has been reported to be 4.6% (2). There is no other published data on tinnitus prevalence and its associated factors in such a sample of the adult population in Iran. In the present study, we reported tinnitus prevalence that lasted more than one week in one or both ears. We also investigated risk factors related to tinnitus in the study population.

## Materials and Methods

This population-based cross-sectional study was based on data from the PERSIAN Guilan Cohort Study (PGCS). The PGCS was conducted on 10520 men and women between 35 and 70 years old in urban and rural areas of “Sowme’e Sara” in Guilan, the northern province of Iran (11). The PGCS was nested within the Iranian Primary healthcare system. At the starting point of the PGCS, trained health workers approached door-to-door in rural and urban areas to inform individuals of the study and its objectives. The contact information of selected individuals was collected, and about 27 to 30 eligible participants were contacted over the phone daily from October 8, 2014, to January 20, 2017. The detail of sampling and data collection methods have been previously described by Poustchi et al. (12).The PERSIAN Cohort questionnaire consists of 482 items, completed with a face-to-face interview and a physical examination accomplished by trained interviewers. Among all of the items in the PGCS, there was one question concerning tinnitus. All of the participants of PGCS were asked if they had tinnitus episodes that lasted at least one week in one or both ears. In the present study, the prevalence of tinnitus has been reported, and its associated non-auditory risk factors and comorbidities have been investigated.Age, sex, habitat, and marital status were collected as demographic variables. Information on employment status was obtained by asking the questions “Are you currently employed?” and “what is the main source of your income?”. Socioeconomic status (SES) was classified into quartiles from Q1 (as the most deprived) to Q4 (as the most privileged) based on questions about “Housing and car ownership status” and "Having home comfort accessories". The participants were asked about their education level and were divided into four groups illiterates, primary education, high school education, and college education. Lifestyle habits, including smoking behavior (nonsmokers vs. smokers who smoked more than 100 cigarettes in a lifetime), alcohol consumption (none or regularly use), body mass index (BMI) as weight in kilograms/height in meters squared, level of physical activity (metabolic equivalent tasks (METs)) as METs/hour/day (13), and sleep duration (≤ 6, 7-8, ≥ 8 hours per day). The presence or absence of hypertension and diabetes were defined according to the PGCS profile (11) and included in the analysis. Other evaluated comorbidities have been reported based on the “number of comorbidities,” including cancer, myocardial infarcts, stroke, epilepsy, chronic headaches, depression, psychiatric disorder, and multiple sclerosis. The study design was approved by the ethics committees at the Guilan University of Medical Sciences (P/3/132/215). Informed consent was obtained from all participants included in this study. 

**Statistical analysis**

This study presented continuous variables as mean ± standard deviation (SD) and categorical variables as number (percentage). Simple and multiple logistic regression analyses were performed to examine the relationship between demographic/clinical factors and tinnitus. The crude and adjusted odds ratio (OR) and 95% confidence interval (CI) were calculated. Statistical analysis was undertaken using SPSS for windows, version 16.0 (SPSS Inc., Chicago, IL, USA), and the significance level was set at 0.05.

## Results

*Characteristics of the participants*

The demographic and clinical characteristics of the participants are presented in Table 1. 

**Table 1:** Demographic and clinical characteristics of the participants in the PERSIAN Guilan Cohort Study (n=10520)

Variable	Total (n=10520)	Male (n=4887)	Female (n=5633)
**Sex**			
**Male**	4887 (46.5)	-	-
**Female**	5633 (53.5)	-	-
**Age (years)**			
**35-44**	3139 (29.8)	1438 (29.4)	1701 (30.2)
**45-54**	3854 (36.6)	1835 (37.3)	2019 (35.8)
**55-64**	2730 (26.0)	1261 (25.8)	1469 (26.1)
**> 65**	797 (7.6)	353 (7.2)	444 (7.9)
**Marital status**			
**Single**	305 (2.9)	78 (1.6)	227 (4.0)
**Married**	9527 (90.6)	4733 (96.8)	4794 (85.1)
**Widow**	566 (5.4)	48 (1.0)	518 (9.2)
**Divorced**	122 (1.1)	28 (0.6)	94 (1.7)
**Education level**			
**Illiterate**	1738 (16.5)	506 (10.4)	1232 (21.9)
**1-5**	3312 (31.5)	1335 (27.3)	1977 (35.1)
**6-12**	4832 (45.9)	2590 (53.0)	2242 (39.8)
**University**	638 (6.1)	456 (9.3)	182 (3.2)
**Employment**			
**Unemployed**	4781 (45.4)	562 (11.5)	4219 (74.9)
**Employed**	5739 (54.6)	4325 (88.5)	1414 (25.1)
**Habitat**			
**Urban**	4613 (43.8)	2062 (42.2)	2551 (45.3)
**Rural**	5907 (56.2)	2825 (57.8)	3082 (54.7)
**BMI (kg/m2)**			
**Normal**	2746 (26.1)	1894 (38.8)	852 (15.1)
**Underweight**	141 (1.3)	110 (2.03)	31 (0.6)
**Overweight**	4198 (39.9)	2095 (42.9)	2103 (37.3)
**Obese**	3435 (32.7)	788 (16.1)	2647 (47.0)
**Smoking**			
**Non-smoker**	7936 (75.4)	2365 (48.4)	5571 (98.9)
**Smoker**	2584 (24.6)	2522 (51.6)	62 (1.1)
**Alcohol consumption**			
**No**	9125 (86.7)	3768 (77.1)	5357 (95.1)
**Yes**	1515 (13.3)	1119 (22.9)	276 (4.9)
**Hypertension**			
**No**	5977 (56.8)	2983 (61.0)	2994 (53.2)
**Yes**	4543 (43.2)	1904 (39.0)	2639 (46.8)
**Diabetes**			
**No**	7989 (75.9)	3898 (79.8)	4091 (72.6)
**Yes**	2531 (24.1)	989 (20.2)	1542 (27.4)
**No. of other diseases**			
**0**	7157 (68.0)	3721 (76.1)	3436 (61.0)
**1**	2373 (22.6)	853 (17.5)	1520 (27.0)
**≥2**	990 (9.4)	313 (6.4)	677 (12.0)
**Sleep time (hour)**			
**≤ 6**	1648 (15.7)	976 (20.0)	672 (11.9)
**7-8**	5399 (51.3)	2681 (54.9)	2718 (48.3)
**≥ 8**	3473 (33.0)	1230 (25.2)	2243 (39.8)

Values are presented as n (%). BMI: Body Mass Index

*Prevalence of tinnitus*

The prevalence of tinnitus was 6.4% in this study and was more prevalent in women than in men (7.0% vs. 5.8%). The prevalence of tinnitus increased with age: the highest was 10.8% in those aged 65 and older (Table 2).

**Table 2:** Prevalence of tinnitus in the PERSIAN Guilan Cohort Study

Sex/Age group	Population (N)	Cases (n)	Prevalence (%)
**Male**			
**35-44**	1438	45	3.1
**45-54**	1835	90	4.9
**55-64**	1261	113	9.0
**≥ 65**	353	34	9.6
**Total**	4887	282	5.8
**Female**			
**35-44**	1701	68	4.0
**45-54**	2019	125	6.2
**55-64**	1469	149	10.1
**≥ 65**	444	52	11.7
**Total**	5633	394	7.0
**Overall**			
**35-44**	3139	113	3.6
**45-54**	3854	215	5.6
**55-64**	2730	262	9.6
**≥ 65**	797	86	10.8
**Total**	10520	676	6.4

*Factors associated with tinnitus using univariable analysis*

Simple logistic regression analysis was applied to identify tinnitus-associated factors (see Table 3). The three older age groups (45–54, 55-64, and >65) were signiﬁcantly more likely than the youngest group (35-44) to have tinnitus; the OR for those aged 45-54 was 1.58 (95% CI: 1.25-2.00), for those aged 45-54 was 2.84 (95% CI: 2.27-3.57), and for those aged 65 and older was 3.24 (95% CI: 2.42-4.34).

Compared to males, females were 1.23 times more likely to have tinnitus than males (OR=1.23, 95% CI: 1.05-1.44). Tinnitus was more prevalent in widow participants than single participants (OR=2.25, 95% CI: 1.25 - 4.04). Illiterate People were 1.71 times more likely to have tinnitus than people with a university education (OR=1.71, 95% CI: 1.19-2.46). Unemployed participants reported higher tinnitus than employed participants (OR=1.54, 95% CI: 1.31 - 1.80). Those residents in rural areas reported higher tinnitus than those in urban areas (OR=1.19, 95% CI: 1.01 - 1.39).

Regarding MET, participants in the first quartile were at significantly increased risk for tinnitus compared to participants in the fourth quartile (OR=1.29, 95% CI: 1.04-1.61). Hypertension increased the likelihood of tinnitus (OR=1.46, 95% CI: 1.25-1.70). Participants with diabetes were 1.38 times more likely to have tinnitus than other participants (OR=1.38, 95% CI: 1.16-1.63). Particpants with one and ≥2 comorbidities had significantly increased odds of tinnitus (OR=2.03, 95% CI: 1.70-2.43 and OR=3.24, 95% CI: 2.62-4.01, respectively) as compared with patients with no comorbidities. The results of subgroup analysis by sex were mainly consistent with the overall analysis in all subjects (Tables 4 and 5).

*Factors associated with tinnitus using multivariable analysis*

Multiple logistic regression analysis was also applied to identify factors associated with tinnitus (Table 3). The adjusted analysis showed that the odds of tinnitus increased with age. The three older age groups (45–54, 55-64, and >65) were signiﬁcantly more likely than the youngest group (35-44) to have tinnitus. The adjusted odds ratios and P-values are presented in Table 3. Those residents in rural areas reported higher tinnitus than those in urban areas (aOR=1.22, 95% CI: 1.03 - 1.44). Cigarette smoking increased the odds of tinnitus (aOR=1.33, 95% CI: 1.04-1.72). Participants with one and ≥2 comorbidities had significantly increased odds of tinnitus (aOR=1.85, 95% CI: 1.54-2.22 and aOR=2.75, 95% CI: 2.19-3.44, respectively) as compared with patients with no comorbidities. Other variables were not significantly associated with tinnitus. In multivariable analysis, the results of subgroup analysis by sex were mainly consistent with the overall analysis in all subjects (Tables 4 and 5).

**Table 3:** Logistic regression analyses for the relationship between demographic/clinical factors and tinnitus among adults (>35 years old) in the PERSIAN Guilan Cohort Study (n=10520)

Variable	Prevalence of tinnitus, n (%)	Unadjusted		Adjusted	
		OR (95% CI)	*P*	OR (95% CI)	*P*
**Age (years)**					
**35-44**	113 (3.6)	1		1	
**45-54**	215 (5.6)	1.58 (1.25 – 2.00)	<0.001	1.48 (1.17 – 1.87)	0.001
**55-64**	262 (9.6)	2.84 (2.27 – 3.57)	<0.001	2.37 (1.85 – 3.02)	<0.001
**> 65**	86 (10.8)	3.24 (2.42 – 4.34)	<0.001	2.60 (1.88 – 3.60)	<0.001
**Sex**					
**Male**	282 (5.8)	1		1	
**Female**	394 (7.0)	1.23 (1.05 – 1.44)	0.011	1.13 (0.86 – 1.48)	0.375
**Marital status**					
**Single**	15 (4.9)	1		1	
**Married**	595 (6.2)	1.29 (0.76 – 2.18)	0.345	1.01 (0.59 – 1.72)	0.985
**Widow**	59 (10.4)	2.25 (1.25 – 4.04)	0.007	1.19 (0.65 – 2.18)	0.580
**Divorced**	7 (5.7)	1.18 (0.47 – 2.96)	0.729	0.99 (0.39 – 2.52)	0.976
**Education level**					
**Illiterate**	170 (9.8)	1.71 (1.19 – 2.46)	0.004	1.09 (0.72 – 1.67)	0.672
**1-5**	187 (5.6)	0.94 (0.66 – 1.35)	0.757	0.85 (0.57 – 1.26)	0.413
**6-12**	281 (5.8)	0.97 (0.69 – 1.38)	0.887	1.00 (0.69 – 1.45)	0.980
**University**	38 (6.0)	1		1	
**Employment**					
**Unemployed**	375 (7.8)	1.54 (1.31 – 1.80)	<0.001	1.22 (0.99 – 1.52)	0.065
**Employed**	301 (5.2)	1		1	
**Habitat**					
**Urban**	270 (5.9)	1		1	
**Rural**	406 (6.9)	1.19 (1.01 – 1.39)	0.034	1.22 (1.03 – 1.44)	0.023
**SES**					
**Q**_1_	184 (7.0)	1.16 (0.93 – 1.45)	0.181	0.96 (0.75 – 1.23)	0.740
**Q**_2_	172 (6.5)	1.08 (0.86 – 1.35)	0.496	0.97 (0.76 – 1.23)	0.778
**Q**_3_	160 (6.1)	1.00 (0.80 – 1.25)	1.000	1.04 (0.82 – 1.32)	0.737
**Q**_4_	160 (6.1)	1		1	
**BMI (kg/m2)**					
**Normal (18.5–24.99)**	170 (6.2)	1		1	
**Underweight (<18.5)**	8 (5.7)	0.91 (0.44 – 1.89)	0.803	0.90 (0.43 – 1.90)	0.791
**Overweight (25–29.9)**	284 (6.8)	1.10 (0.90 – 1.34)	0.344	1.07 (0.87 – 1.31)	0.521
**Obese (≥30)**	214 (6.2)	1.01 (0.82 – 1.24)	0.949	0.93 (0.93 – 1.17)	0.560
**Physical activity** **(METs/hour/day)**					
**Q1 (<34.8)**	191 (7.3)	1.29 (1.04 – 1.61)	0.022	0.90 (0.70 – 1.16)	0.434
**Q2 (34.8-38.8)**	173 (6.6)	1.16 (0.93 – 1.46)	0.187	0.94 (0.73 – 1.20)	0.597
**Q3 (38.8-45.8)**	162 (6.2)	1.09 (0.86 – 1.36)	0.484	0.95 (0.75 – 1.21)	0.698
**Q4 (≥45.8)**	150 (5.7)	1		1	
**Smoking**					
**Non-smoker**	497 (6.3)	1		1	
**Smoker**	179 (6.9)	1.11 (0.93 – 1.33)	0.232	1.33 (1.04 – 1.72)	0.025
**Alcohol consumption**					
**No**	586 (6.4)	1		1	
**Yes**	90 (6.5)	1.00 (0.80 – 1.26)	0.968	0.86 (0.66 – 1.11)	0.236
**Hypertension**					
**No**	325 (5.4)	1		1	
**Yes**	351 (7.7)	1.46 (1.25 – 1.70)	<0.001	1.06 (0.90 – 1.26)	0.490
**Diabetes**					
**No**	474 (5.9)	1		1	
**Yes**	202 (8.0)	1.38 (1.16 – 1.63)	<0.001	1.02 (0.85 – 1.22)	0.835
**No. of other diseases**					
**0**	330 (4.6)	1		1	
**1**	212 (8.9)	2.03 (1.70 – 2.43)	<0.001	1.85 (1.54 – 2.22)	<0.001
**≥2**	134 (13.5)	3.24 (2.62 – 4.01)	<0.001	2.75 (2.19 – 3.44)	<0.001
**Sleep time (hour)**					
**≤ 6**	108 (6.6)	1		1	
**7-8**	348 (6.4)	0.98 (0.79 – 1.23)	0.876	1.01 (0.80 – 1.27)	0.944
**≥ 8**	220 (6.3)	0.96 (0.76 – 1.22)	0.765	0.93 (0.72 – 1.19)	0.561

BMI: Body Mass Index; OR: Odds Ratio; CI: Confidence Interval

**Table 4:** Logistic regression analyses for the relationship between demographic/clinical factors and tinnitus among male adults (>35 years old) in the PERSIAN Guilan Cohort Study

Variable	Prevalence of tinnitus, n (%)	Unadjusted		Adjusted	
		OR (95% CI)	*P*	OR (95% CI)	*P*
**Age (years)**					
**35-44**	45 (3.1)	1		1	
**45-54**	90 (4.9)	1.60 (1.11 – 2.30)	0.012	1.36 (0.94 – 1.97)	0.106
**55-64**	113 (9.0)	3.05 (2.14 – 4.34)	<0.001	2.13 (1.44 – 3.15)	<0.001
**> 65**	34 (9.6)	3.30 (2.08 – 5.24)	<0.001	2.15 (1.27 – 3.63)	0.004
**Marital status**					
**Single**	2 (2.6)	1		1	
**Married**	275 (5.8)	2.34 (0.57 – 9.59)	0.236	1.96 (0.47 – 8.22)	0.357
**Widow**	3 (6.2)	2.53 (0.41 – 15.74)	0.319	1.14 (0.18 – 7.37)	0.890
**Divorced**	2 (7.1)	2.92 (0.39 – 21.81)	0.296	2.54 (0.33 – 19.74)	0.372
**Education level**					
**Illiterate**	49 (9.7)	1.93 (1.16 – 3.20)	0.011	1.64 (0.90 – 2.98)	0.104
**1-5**	55 (4.1)	0.77 (0.47 – 1.26)	0.306	0.86 (0.50 – 1.49)	0.594
**6-12**	154 (5.9)	1.14 (0.73 – 1.77)	0.567	1.37 (0.85 – 2.20)	0.196
**University**	24 (5.3)	1		1	
**Employment**					
**Unemployed**	70 (12.5)	2.76 (2.07 – 3.67)	<0.001	1.79 (1.27 – 2.53)	0.001
**Employed**	212 (4.9)	1		1	
**Habitat**					
**Urban**	114 (5.5)	1		1	
**Rural**	168 (5.9)	1.08 (0.85 – 1.38)	0.536	1.03 (0.78 – 1.34)	0.848
**SES**					
**Q**_1_	64 (6.3)	1.07 (0.76 – 1.49)	0.711	0.83 (0.56 – 1.25)	0.376
**Q**_2_	66 (5.8)	0.96 (0.69 – 1.34)	0.822	0.83 (0.57 – 1.20)	0.316
**Q**_3_	68 (5.1)	0.85 (0.61 – 1.18)	0.342	0.84 (0.59 – 1.19)	0.323
**Q**_4_	84 (6.0)	1		1	
**BMI (kg/m2)**					
**Normal**	112 (5.9)	1		1	
**Underweight**	7 (6.4)	1.08 (0.49 – 2.38)	0.846	0.92 (0.41 – 2.08)	0.843
**Overweight**	122 (5.8)	0.98 (0.76 – 1.28)	0.904	1.02 (0.77 – 1.34)	0.908
**Obese**	41 (5.2)	0.87 (0.60 – 1.26)	0.470	0.93 (0.63 – 1.36)	0.691
**MET**					
**Q**_1_	80 (7.6)	1.48 (1.08 – 2.01)	0.013	0.94 (0.65 – 1.35)	0.738
**Q**_2_	51 (5.5)	1.06 (0.74 – 1.50)	0.757	0.84 (0.58 – 1.23)	0.374
**Q**_3_	57 (5.1)	0.96 (0.68 – 1.34)	0.801	0.87 (0.61 – 1.23)	0.431
**Q**_4_	94 (5.3)	1		1	
**Smoking**					
**Non-smoker**	111 (4.7)	1		1	
**Smoker**	171 (6.8)	1.48 (1.16 – 1.89)	0.002	1.18 (0.90 – 1.56)	0.232
**Alcohol consumption**					
**No**	206 (5.5)	1		1	
**Yes**	76 (6.8)	1.26 (0.96 – 1.65)	0.096	1.03 (0.76 – 1.40)	0.829
**Hypertension**					
**No**	151 (5.1)	1		1	
**Yes**	131 (6.9)	1.39 (1.09 – 1.76)	0.008	0.99 (0.76 – 1.29)	0.922
**Diabetes**					
**No**	211 (5.4)	1		1	
**Yes**	71 (7.2)	1.35 (1.02 – 1.79)	0.034	1.03 (0.76 – 1.39)	0.848
**No. of other diseases**					
**0**	159 (4.3)	1		1	
**1**	72 (8.4)	2.07 (1.55 – 2.76)	<0.001	1.84 (1.37 – 2.48)	<0.001
**≥2**	51 (16.3)	4.36 (3.11 – 6.12)	<0.001	3.37 (2.33 – 4.85)	<0.001
**Sleep time (hour)**					
**≤ 6**	52 (5.3)	1		1	
**7-8**	152 (5.7)	1.07 (0.77 – 1.48)	0.691	1.11 (0.80 – 1.55)	0.538
**≥ 8**	78 (6.3)	1.20 (0.84 – 1.73)	0.316	1.13 (0.78 – 1.64)	0.523

BMI: Body Mass Index; OR: Odds Ratio; CI: Confidence Interval

**Table 5:** Logistic regression analyses for the relationship between demographic/clinical factors and tinnitus among female adults (>35 years old) in the PERSIAN Guilan Cohort Study

Variable	Prevalence of tinnitus, n (%)	Unadjusted		Adjusted	
		OR (95% CI)	*P*	OR (95% CI)	*P*
**Age (years)**					
**35-44**	68 (4.0)	1		1	
**45-54**	125 (6.2)	1.58 (1.17 – 2.15)	0.003	1.49 (1.10 – 2.03)	0.011
**55-64**	149 (10.1)	2.71 (2.02 – 3.64)	<0.001	2.32 (1.69 – 3.20)	<0.001
**> 65**	52 (11.7)	3.19 (2.18 – 4.65)	<0.001	2.78 (1.83 – 4.24)	<0.001
**Marital status**					
**Single**	13 (5.7)	1		1	
**Married**	320 (6.7)	1.18 (0.67 – 2.08)	0.575	0.88 (0.49 – 1.59)	0.674
**Widow**	56 (10.8)	2.00 (1.07 – 3.73)	0.030	1.11 (0.58 – 2.15)	0.751
**Divorced**	5 (5.3)	0.92 (0.32 – 2.67)	0.885	0.73 (0.25 – 2.17)	0.573
**Education level**					
**Illiterate**	121 (9.8)	1.31 (0.73 – 2.33)	0.363	0.80 (0.42 – 1.53)	0.493
**1-5**	132 (6.7)	0.86 (0.48 – 1.52)	0.602	0.69 (0.37 – 1.29)	0.250
**6-12**	127 (5.7)	0.72 (0.41 – 1.28)	0.263	0.67 (0.37 – 1.24)	0.201
**University**	14 (7.7)	1		1	
**Employment**					
**Unemployed**	305 (7.2)	1.16 (0.91 – 1.48)	0.233	1.00 (0.77 – 1.31)	0.989
**Employed**	89 (6.3)	1		1	
**Habitat**					
**Urban**	156 (6.1)	1		1	
**Rural**	238 (7.7)	1.28 (1.04 – 1.58)	0.019	1.38 (1.11 – 1.72)	0.004
**SES**					
**Q**_1_	120 (7.4)	0.96 (0.73 – 1.26)	0.777	1.04 (0.75 – 1.46)	0.798
**Q**_2_	106 (7.1)	0.95 (0.71 – 1.26)	0.707	1.08 (0.78 – 1.51)	0.630
**Q**_3_	92 (7.0)	0.83 (0.62 – 1.12)	0.214	1.23 (0.88 – 1.70)	0.223
**Q**_4_	76 (6.2)	1		1	
**BMI (kg/m2)**					
**Normal**	58 (6.8)	1		1	
**Underweight**	1 (3.2)	0.46 (0.06 – 3.41)	0.444	0.54 (0.07 – 4.12)	0.551
**Overweight**	162 (7.7)	1.14 (0.84 – 1.56)	0.401	1.17 (0.85 – 1.60)	0.347
**Obese**	173 (6.5)	0.96 (0.70 – 1.30)	0.781	0.99 (0.72 – 1.36)	0.959
**MET**					
**Q**_1_	111 (7.0)	1.07 (0.77 – 1.49)	0.697	0.85 (0.59 – 1.23)	0.384
**Q**_2_	122 (7.1)	1.08 (0.78 – 1.50)	0.638	0.97 (0.68 – 1.37)	0.848
**Q**_3_	105 (7.0)	1.06 (0.76 – 1.48)	0.738	0.98 (0.69 – 1.38)	0.904
**Q**_4_	56 (6.6)	1		1	
**Smoking**					
**Non-smoker**	386 (6.9)	1		1	
**Smoker**	8 (12.9)	1.99 (0.94 – 4.21)	0.072	1.78 (0.81 – 3.89)	0.151
**Alcohol consumption**					
**No**	380 (7.1)	1		1	
**Yes**	14 (5.1)	0.70 (0.41 – 1.21)	0.202	0.49 (0.28 – 0.87)	0.014
**Hypertension**					
**No**	174 (5.8)	1		1	
**Yes**	220 (8.3)	1.47 (1.20 – 1.81)	<0.001	1.12 (0.90 – 1.41)	0.317
**Diabetes**					
**No**	263 (6.4)	1		1	
**Yes**	131 (8.5)	1.35 (1.09 – 1.68)	0.007	1.04 (0.82 – 1.31)	0.772
**No. of other diseases**					
**0**	171 (5.0)	1		1	
**1**	140 (9.2)	1.94 (1.54 – 2.44)	<0.001	1.85 (1.46 – 2.34)	<0.001
**≥ 2**	83 (12.3)	2.67 (2.02 – 3.52)	<0.001	2.40 (1.80 – 3.20)	<0.001
**Sleep time (hour)**					
**≤ 6**	56 (8.3)	1		1	
**7-8**	196 (7.2)	0.85 (0.63 – 1.17)	0.321	0.91 (0.67 – 1.25)	0.575
**≥ 8**	142 (6.3)	0.74 (0.54 – 1.03)	0.071	0.78 (0.56 – 1.09)	0.152

BMI: Body Mass Index; OR: Odds Ratio; CI: Confidence Interval

## Discussion

*Prevalence of tinnitus*

This investigation was the first epidemiologic study in Iran that investigated the prevalence of tinnitus and its associated factors based on representative data from a cohort study. Several population-based studies have reported the prevalence of tinnitus in different countries. A study in the Italian population reported a prevalence rate of 14.5% for subjective tinnitus (14). The prevalence of tinnitus was 16.9%  in a large sample of adults aged 40 to 69 years from the United Kingdom (15).A study on older than 60 individuals in Brazil reported a prevalence of 42.77% of tinnitus (16). In a large sample of 99,435 individuals older than 20 in the United States, the prevalence of tinnitus was reported as 5.4% (17). The prevalence of tinnitus was 18.6% in a study of Japanese Elders (9). In the South East Asia, an Egyptian community-based survey involving 8,484 subjects has reported a prevalence of 5.17% in adults over 18 years old (18). In a study on the 3207 Iranian population, the prevalence of tinnitus has been reported to be 4.6% among urban individuals (2).

Different characteristics of the surveyed population or definitions of tinnitus have led to different prevalence rates. A total of 10520 men and women older than 34 from urban and rural areas of “Sowme’e Sara” country in northern Iran were included in the present study. In this cohort study, a yes/no response was used to identify tinnitus that lasted for one week or more in one or both ears. These data were collected with a face-to-face interview. The overall prevalence of tinnitus was reported to be 6.4%. A direct comparison between this prevalence data and those from other studies is difficult. What is evident is the noticeable prevalence of tinnitus in the study population that was not associated with marital status, educational level, employment, socioeconomic status (SES), alcohol consumption, BMI, level of physical activity, sleep duration, hypertension, and diabetes. When compared between sex groups by adjusted analysis, there was also no effect of gender in the prevalence of tinnitus between the two groups. There are different reports regarding sex differences in the prevalence of tinnitus in the literature. Some of them reported no difference between genders (2, 9, 18), a few reported as much as a two-fold risk of tinnitus in men than in women and (19), and some have reported a higher prevalence of tinnitus in females (3, 10, 20). It seems that a meta-analysis of the literature is needed to identify the association between the variables mentioned above and the prevalence of tinnitus. 

*Factors associated with tinnitus*

The relationship between demographic and non-auditory clinical factors with tinnitus has been investigated in this study. According to the study results, tinnitus was more frequently reported in older participants than the younger ones. About 5.6% of participants in the 45-54 years old age group, 9.6% of participants in the 55-64 years old age group, and 10.8 over 65 years old individuals had reported having tinnitus. In comparison, tinnitus was reported in only 3.6% of participants in the 35-44 age group. The adjusted logistic regression analyses showed that the three older age groups were signiﬁcantly more likely to have tinnitus than the youngest group. Since previous research has suggested that both age and tinnitus are related to hearing (21, 22), This finding which follows many previous reports (10, 18, 23, 24), may be due to increased odds of hearing impairment with increasing age (15). 

We found that, in our study population, habitat can be associated with tinnitus. In the present study, those who were in rural habitats reported tinnitus more frequently than those who were residents in urban areas. Some of the previous studies have reported more prevalence of tinnitus in urban areas, and some found tinnitus to be more prevalent in rural areas. In an Egyptian epidemiological study of chronic tinnitus on 5,783 subjects from the rural and 2,701 subjects from the urban community, Khedr stated that the prevalence rate of tinnitus was significantly higher among urban than rural (18). Xu and colleagues reported that in individuals with or without hearing impairment, tinnitus was more prevalent in rural areas (25). It is speculated that limited knowledge of health and inferior access to medical support makes the living condition worse in rural areas and may have a role in more common otologic problems like tinnitus. Although a handful of reports regarding the association between tinnitus and smoking (3, 20, 26), such a finding is inconsistent in the literature. For instance, Park reported no association between tinnitus prevalence and past or present smoking history in South Korea in 21,893 individuals over 12 years of age (10). In the present study, cigarette smoking was associated with tinnitus. Individuals with a history of smoking had a significantly higher prevalence tinnitus. Previous studies have stated the cigarette smoking is a risk factor for hearing loss and cochlear dysfunction (27, 28), which can explain why tinnitus is more prevalent in cigarette smokers. Whether this is a result of a hearing problem or because of more vulnerability of adolescents with tinnitus to substance abuse (3) needs more investigation.

In the present study, although individuals with hypertension and diabetes reported tinnitus more frequently, these conditions could not predict tinnitus in the adjusted analysis. The reports of the relationship between hypertension and diabetes with tinnitus have been controversial. Similar results were reported by Kim et al. (29); hypertension and diabetes did not increase the odds of frequent tinnitus in 14,178 participants in the 1999-2004 National Health and Nutrition Examination Surveys (30), whereas in the study of Nondahl et al. neither hypertension nor diabetes was associated with having tinnitus (31). 

Tinnitus is a multifactorial symptom, and many clinical conditions, somatic disorders, and pharmaceutical drugs can induce it (24). According to previous reports, depression and psychiatric disorders, multiple sclerosis, myocardial infarcts, stroke, epilepsy (34), chronic headaches, and different types of cancer can be considered as risk factors for tinnitus (9,31-36). When these comorbidities were investigated in this study, a significant association was observed between tinnitus and having one or more comorbidity. Many of these conditions may directly or indirectly influence the auditory system or neural networks involved in the perception of tinnitus which could not be assessed in our study. Future work should find the cause-and-effect relationship between tinnitus and such risk factors. 

There were limitations in the present study. First, the data on tinnitus were collected through a self-reported questionnaire, and there was no additional information about tinnitus characteristics in the study population. Second, need for more data about the hearing thresholds of the participants in the present study made it difficult to discuss the association between tinnitus and factors like habitat, smoking, and the number of comorbidities. These limitations should be noticed when interpreting our findings. 

## Conclusion

Our results revealed that the prevalence of tinnitus in north Iran is about 6.4% and comparable with other communities. In agreement with previous literature, these results show that the causes of tinnitus can be diverse. Therefore, in planning for treating and managing tinnitus, attention should be paid to the widespread causes and comorbidities.
